# ‘Bellography’: Life and Contributions of Ross and Joyce Bell, two New England Naturalists

**DOI:** 10.3897/zookeys.147.1999

**Published:** 2011-11-16

**Authors:** John R. Spence, George E. Ball, Robert L. Davidson, Jessica J. Rykken

**Affiliations:** 1 Department of Renewable Resources, 4-42 Earth Sciences Bldg., University of Alberta, Edmonton, AB, Canada T6G 2E3; 2 Department of Biological Sciences, CW-405 Biological Sciences Bldg., University of Alberta, Edmonton, AB, Canada T6G 2E9; 3 Carnegie Museum of Natural History, 4400 Forbes Avenue, Pittsburgh, PA 15213, USA; 4 Museum of Comparative Zoology, Harvard University, 26 Oxford Street, Cambridge, MA 02138, USA

**Keywords:** Ross Bell, Joyce Bell, Carabidae, Rhysodini, Vermont

## Abstract

The lives and contributions of Ross and Joyce Bell are described with particular attention to studies of invertebrate natural history in the state of Vermont and carabid beetles of several groups, including the world rhysodine fauna. Their work, all done at the University of Vermont, was mainly taxonomic in nature and included aspects of the biology of the species considered. During their careers they described more than 75% of the c. 340 rhysodine species known to science. Ross Bell also wrote a number of seminal papers about the basal relationships of the Adephaga and the comparative anatomy of carabid coxal cavities. Ross and Joyce inspired several generations of students at UVM to take up advanced work in entomology and natural history.

Preceding production of this Festschrift, long-time friends and associates of Joyce and Ross Bell assembled at the University of Vermont (UVM) in Burlington during 10-13 June 2010, to honor them and recognize their contributions to local natural history in particular, to systematic entomology in general. In the printed Festschrift it is appropriate to provide a brief summary of their lives and their work in entomology. Ross Taylor Bell was born in Champaign, Illinois, on 23 April 1929. His father, Alfred Hannam Bell, was from Ontario, Canada, and studied Geology at the Universities of Toronto (B.Sc.) and Chicago (Ph.D.). He was an influential and productive petroleum geologist with the Illinois Geological Survey in Urbana. Alfred Bell’s second wife, Dorothy Becker of Cincinnati, Ohio, was also a geologist employed at the Survey, having received her B.S. in Geology at the University of Cincinnati. Ross’ parents met after the early death of Alfred’s first wife from tuberculosis. They had three children, Ross and his two sisters Martha and Enid. By all accounts they were a family of enthusiastic naturalists. Both Martha and Enid married students who were contemporaries of Ross in the Department of Zoology at the University of Illinois and, furthermore, Martha became a professional botanist. Family vacations, which included long drives to various points in North America, were invariably a mix of botany, zoology and geology, including all the ‘stuff’ of classical natural history.

Ross Bell’s interest in insects began with the childhood gift of an insect collecting kit from his parents. This interest was exacerbated when someone gave him a jar full of silk worm larvae, which he reared on mulberry leaves, incidentally he claims developing his skills as a tree climber. At age 14, Ross got a job at the Natural History Survey at the University of Illinois. In this capacity he trapped flies and rose to the challenge of sorting sarcophagids, muscids and calliphorids and identifying them. The next summer he accepted a position with the taxonomic survey that he thought would take him toward his new found passion for insect identification, but instead he ended up washing glassware, installing moth balls in the collection and gathering box elder leaves for some reason that can no longer be recollected. Because this was during WW II, Ross posits that his eclectic roster of tasks resulted from a shortage of grad students for ‘slave labor’. Very likely these experiences had something to do with the respectful way that he subsequently treated his own students, always encouraging and supporting them to follow their own noses forward, knowing all along that a professor was interested in both them and their scientific findings.

Ross went to High School at the University Laboratory High School in Champaign, which he recalls as a magnificent place -- and so it must have been because it produced three Nobel Prize Laureates, two of them in science. Ross’s mother always thought that he should’ve done the same, but alas, as Ross pointed out in self-defense, no such prize was awarded in the category of ‘carabidology’. Ross spent the halcyon summers of his youth at his Aunt and Uncle’s farm in West Alexandria, Ohio, helping with farm chores, and at every chance collecting and attempting to name insects from the fields and a stream which bordered the east boundary of the farm. This was his first bash with aquatic insects, something that remained a side interest throughout his career. In his last year of high school he rode his balloon-tired bicycle all the way to West Alexandria. It required two days of pedaling and an overnight at a hotel in Noblesville, Indiana. This 215 mile bicycle ride was an achievement that gave Ross much pleasure, both in the doing and in the subsequent telling.

Ross spent the immediate post-war years (1946-49) at the University of Illinois where he earned a B.S. in Zoology. In one additional year, he earned an M.S. with a thesis about the Carabidae (Simplicia) of Illinois. Between 1950-53 he completed a doctoral dissertation about the comparative morphology and phylogeny of Adephaga, under the sponsorship of the well-known entomologist, W. V. Balduf, whose early pioneering work in ‘bionomics’ helped establish the scientific basis for insect ecology in North America. Balduf introduced Ross to Blatchley’s Coleoptera of Indiana in exchange for Ross’s efforts in sorting and counting rose hips for one of Balduf’s studies. Ross became much interested in the proper application of names through exposure to the plant taxonomist E. Neville Jones. With this motivation, Blatchley’s useful keys allowed Ross to confidently name his catches and lured him into the world of beetles (e.g., Bell 1955, 1957, 1958, 1959, 1960). His resulting thesis work on the Adephaga is recognized as a classic among carabidologists and it led to a host of publications on the topic (Bell 1964, 1965, 1966a, 1967). One of Ross’s best friends during his time at Illinois was “Butterfly” Bob Snetsinger, who became an economic entomologist at Penn State. Spurred on by interactions with Bob, Ross developed interests in life history and larval biology (Bell 1990, 1991), and passed them on enthusiastically to his students (e.g., Spence et al. 1976). While at Illinois, Ross also became interested in ecology, was inspired by Professors Victor Shelford, S. Charles Kendeigh and Arthur Vestal, and served as President of the department’s Ecology Club.

At graduation and with Ph.D. in hand, Ross was awarded a Fulbright Fellowship to go to India. After receiving his boat ticket he was packing to sail in two weeks when he received a letter from the Selective Service Board ordering him to appear instead for military duty. Thus he got an unexpected all-expense paid trip to Fort Leonard Wood in the winter for basic training. After his basic training he was sent to Fort Dietrich, MD, then known as the nation’s ‘Germ Warfare Center’. Here he spent two years in the company of fleas. He regards his main accomplishment as discovery of a fast way of distinguishing males from females during the sorting of samples; males wear pinstripes, females do not.

 Upon discharge from the US Army’s flea sorting brigade in 1955, Ross was hired by the Department of Zoology at the University of Vermont to fill a one-year position. The University realized that they had a ‘catch’ and offered him a permanent tenured position a year later. He spent his entire career there as a popular professor contributing excellent service to the University and teaching inspirational courses in Field Zoology, Invertebrate Zoology, Entomology and a summer course for graduate students in Mountain Ecology. During this period he inspired numerous undergraduate students, guided seven graduate students over the course, pursued taxonomic and natural history research on the New England and Vermont faunas and studied rhysodine beetles on a worldwide basis. For most of his career Ross was the Zoology Department’s main strength in systematics and natural history as it connected to ecology and evolutionary biology.

Shortly after joining UVM, Ross began a program of summer collecting followed by taxonomic work that would characterize his research career. He spent the summer of 1956 collecting insects in Mexico with Don Van Horn, a graduate student companion at the University of Illinois, who had gone on to teach entomology at the University of Colorado. It was on this trip that Ross found his first undescribed rhysodine beetle species, and so began a thread of passionate discovery that established him as the world’s expert on these interesting beetles (Bell 1970, 1973, 1975, 1977, 1979, 1985a,b, 1998, 1999; Bell and Bell 1962, 1975, 1978, 1979, 1981, 1982, 1987a,b,c, 1988, 1989b, 1991, 1993, 1995, 2000, 2002, 2009, 2010, 2011). In the following year Ross collected a new entomological companion through marriage to Joyce Elaine Rockenbach of Whitestone, Queens, New York City. Joyce, who had completed her B.S. at Queen’s College, had come to UVM to pursue a Master’s degree after eight years of being a research assistant at the Columbia College of Physicians and Surgeons. Interestingly, Joyce’s graduate advisor at UVM was Reuben Torch, who had been Ross’ assistant lab instructor at the University of Illinois. After ten years of teaching nurses in the UVM School of Nursing, Joyce turned her fulltime attention to entomological pursuits, jointly with Ross. They became inseparable companions in the pursuit of entomology -- especially of carabid lore – and through these endeavors also became well-known pillars of the Vermont naturalists community. As Ross’ eyesight waned, Joyce became his eyes and illustrator for the taxonomic work. Her skills as an excellent microscopist were much appreciated by Ross and his students. Although her grandfather had told her that ‘a woman can either teach or be a nurse’, Joyce Bell became an excellent entomologist in her own right.

The newlyweds went on a honeymoon ‘collecting’ trip to Bar Harbor, during which they recall that they spent more time making car repairs than collecting beetles. The following summer they collected on Cape Breton, Nova Scotia, and also in the Great Smoky Mountains, where they met career-long ‘beetle buddies’ Tom Barr and Willy Rosenberg. He and Joyce returned to Mexico in the summer of 1959, driving from Burlington to Chiapas where they stayed with Franz Blom in San Cristobal. Franz was an archeologist-anthropologist, who worked with the indigenous people of the area, and his wife, Trudy, was a Swiss photographer studying the Lacondon Indians of the forests in the area. Franz had purchased a monastery and used it as his home and research base. He generously took in grad students, mainly in anthropology, and mainly from the University of Michigan, but also opened his doors to other people who wanted to study in the region. A stay in the monastery came with room and board and free evening fireside chats. The food, fondly recalled as ‘tasty Mexican dishes, including memorable stuffed squash blossoms’, came mainly from the Blom garden. During their return to Vermont, Ross and Joyce stopped in Arizona near Nogales in the Chiricahua Mountains where they met for the first time George and Kay Ball, their 2 sons and Ron Madge. So began another career-long association and beetle intensive interaction.

During the 1960s the Bells began a program to learn the fauna of Vermont and to compile extensive records of natural history information. Their goal, as explained to us, was to provide a scientific basis for the study of arthropods in Vermont and to facilitate proper identification of insect specimens. Ross and Joyce developed a system of establishing a base camp of operation in an area and then collecting extensively in that general locale. For example, over a span of eight years they rented ‘summer houses’ around Vermont. During three summers spent in Stowe, they collected extensively on and around Mt. Mansfield, the state’s highest mountain. They also spent summers collecting in northeastern Vermont around Lake Willoughby, in southern Vermont based near Manchester and at other locations in the state. Through this work they built the UVM Entomological Collection into a significant resource for northern New England. In Victory Bog on the Moose River near Lake Willoughby, Andy Moldenke and the Bells first collected *Bembidion bellorum*, eventually described and named in their honor by David Maddison.

The Bells frequently ran light traps during these summer evenings, and this activity contributed much to the excitement of the chase. One night, for example, the light trapping garnered three specimens of *Platypatrobus lacustris*. This was an exciting find because the beetle was known from only two puzzling specimens (one from the shores of Lake Superior, the other taken in a Maine light trap) and its habits were virtually unknown. Once advised of this capture, Phil Darlington and his wife, Libby, drove immediately from Harvard up to Vermont and they all invested much energy in searching for the habitat of this rare catch. Unfortunately they were not successful; however, the story did not end there, but instead the mystery was solved through the web of carabidological connections in which Ross and Joyce were enmeshed, as elaborated below.

Pursuit of their ‘naturalist’ activities led to numerous intersections between the Bells and others of entomological persuasion, especially those interested in ground beetles. These connections were of great benefit, interest and inspiration to Ross and his students. For example, in 1967 Carl Lindroth, the famous Swedish entomologist and carabid aficionado, visited the Bells and gave a talk at UVM about his work on the faunal development of Surtsey Island, Iceland, after its then recent volcanic uprising from the ocean floor. This drew three Quebec entomologists to Vermont and provided the first connection between the Bells and André Larochelle and Henri Goulet. Only later, however, did Henri Goulet discover that *Platypatrobus lacustris* lived in beaver houses. Despite having extensively searched the beaver meadow near the light trap location, the Bells and Darlingtons had not thought to disassemble the house itself and so the discovery eluded them. During the Stowe summers Ross and Joyce also met Ken Cooper, then at Dartmouth. Although a wasp specialist, Ken was also interested in carabids, and a number of techniques that Ken developed, including methodology for everting the internal sac of carabid males, made their way into the playbooks used by Ross and his students. During a summer spent in southern Vermont, they met Carl Parsons, an ex-UVM professor who had abandoned academe to open a bookstore in Manchester. Parsons was an avid collector who willed his excellent Vermont collection to the University, and thus the room that houses the entomological collections in historic Torrey Hall at UVM was named in his honor.

Although this valuable aspect of the careers of academics is frequently underestimated, interested people with active research programs become the nodes of scholarly interaction that link and encourage development of research communities. Ross and Joyce Bell were for many years a significant link in North America’s carabidological community, and a central node for entomology in the State of Vermont. They have been mainstays of the Vermont Entomological Society (VES) founded in 1992. Ross served as one of the first Presidents of the VES, and the Bells participated in the society’s summer field trips and provided much input for the VES Newsletter. This newsletter has become a heartbeat for entomology in Vermont and it is edited by Trish Hanson, one of Ross’ graduate students and who is now an entomologist with the Vermont Forest, Parks and Recreation Department.

The 1970’s and 1980’s were very busy but stimulating years for Ross and Joyce, partly because they extended the geographical boundaries for their entomological work beyond Vermont. For example, in 1970 Ross was hired for the summer to work on the NSF ‘Biome Project’ on the short grass prairie of Pawnee, Colorado. Ross produced keys to Carabidae of the area (Bell 1971) as well as a key to the darkling beetles of the area. In 1974 the Bells joined newly-hired UVM colleague and mammalogist Charles Woods on a trip to Haiti. While Charles looked for mammals, the Bells enriched their rhysodine collection and were drawn further into the mysterious tunnels of rhysodine biology. In 1982 Ross had a sabbatical which took Joyce and he to New Zealand and Papua New Guinea, where they worked out of the Wau Ecological Institute in the northeastern New Guinea Highlands. They were introduced to the Institute by Lindsay and Peg Gressitt of the Bernice P. Bishop Museum, who were killed tragically later that year in a plane crash in China. In addition to fending off burglaries by natives, they spent many days climbing Mt. Missim searching for rhysodines and other entomological treasures. One of those rhysodines was an undescribed species, named by Ross as *Omoglymmius* (s. str.) *gressitti* in honor of Gressitt. Their ‘guard’ at Wau was a reformed cannibal who sported a bone through his nose and carried a spear, and Joyce spent many a sleepless night fearing that the guard might have a relapse in habit.

 In 1989-90 Ross took a second sabbatical during which he and Joyce were ‘down under’ in Australia, this time headquartered in Canberra with the CSIRO (Commonwealth Scientific & Industrial Research Organization). During this trip they undertook two extensive collecting trips in search of rhysodines, one to Tasmania and the other to Queensland. The brilliant carabidologist, Barry Moore, was their host, friend and tour guide for the year. Ross and Joyce fondly recall his enormous contributions to their understanding of Australian natural history. Upon their return from the last trip to Australia, Jonathan Leonard proposed that he and Ross should write a book on Tiger beetles of the Northeastern United States. Thus, the “Northeastern Tiger Beetles” (Leonard and Bell 1999) was born as the definitive reference to these beetles, with color plates of all the species and including keys for third instar larval identification.

Students at UVM were attracted to entomological work because of Ross’ informative teaching style, filled with anecdotes about the doing of natural history, and his clear philosophy of helping others learn how to construct and pursue their own dreams. He was among a handful of professors at UVM during those times whose research work connected to field biology and natural history, and thus was a lightning rod for many students interested in these areas. The Bells generously involved many students in their summer research adventures. For example, the late Bob Mills, who was an accomplished Biology teacher at Putney School, was an able, enthusiastic assistant during the summers in Stowe. Mike Bouffard also worked with the Bells as an undergraduate student. He and Reni Shangraw, another student who worked in Biology with Ross, married and immigrated to Tasmania where Mike took a job teaching High School Biology in Huonville. Mike was recently awarded Australia’s highest award for high school teaching and remains active as a buprestid enthusiast and collector, contributing among other things Tasmanian carabids of interest to the work of Bob Davidson at CMNH – a further example of continuing entomological threads starting with the Bells. Students fortunate to be part of the summer collecting blitzes learned the fascination of finding new things and learning to put names on them effectively. In the big picture they also learned about how such activities form the basis for zoological research that connects to what people can observe directly in their own backyards. There were ancillary lessons; e.g., that gin and tonic is not just some high-brow concoction, but also a great pre-dinner drink on a sultry July evening, or that Joyce Bell was an excellent and adventurous cook, in addition to having rare talents for making unusual entomological discoveries!

Many of the things that Ross pursued were eventually connected to – or even expanded in – work done by his students. For example, Bart Chiolino, a promising Vermont student, worked as Ross’ assistant during the summer in Colorado, before completing an M.S. on wing-dimorphism in ground beetles. During the summer spent in the area of Lake Willoughby they were joined by Andy Moldenke, a summer research student on leave from Wesleyan College. Moldenke subsequently earned a doctorate in entomology at Stanford University and spent a productive career as a soil biologist at Oregon State University. Bob Davidson’s extensive treatments of *Chlaenius* also flowed downstream from work that Ross had done (Bell 1958, 1960, 1966b). Bob, who began as an English major at UVM, was coaxed into entomology through exposure to Ross’ course, and began to collect and study carabids as an undergraduate. Encouraged by the Bells, he continued to collect during his three years in the Peace Corps in Nepal (many new carabids subsequently described), and during this time was invited to return to UVM as Ross’ graduate student in entomology, which led to a successful career as a coleopterist and curator at the Carnegie Museum of Natural History. John Spence’s Master’s work on *Nebria* grew out of a discussion that linked Ross’ first publication (Bell 1955) to observations of the day on the rocky banks of Gleason Brook. This work and Denise Martin Leonard’s M.S. project about stoneflies involved periodic visits to many stations along the length of the stream at different elevations, and through active participation in these research projects, Ross and Joyce came to know virtually every inch of ‘the Brook’. In fact, Gleason Brook and the adjacent North-facing slopes of Camel’s Hump loom large in Ross’ life as a special area for both teaching and research. Other students, including Brian Farrell, Jonathan Leonard, John Strazanac, and Peter Wimmer participated in the ‘Bell natural history blitzes’ and went on to successful careers in entomology.

Ross’ Invertebrate Zoology course became especially popular with graduate students in the Field Naturalist Program at UVM in the 1990s, and this experience inspired Jessica Rykken, for example, to study the role of carabids as indicators of land types in the Green Mountains. She then followed the Bell network west to study carabids and other invertebrates in riparian areas with Andy Moldenke, and later with Brian Farrell undertook an all-taxon biotic inventory of the Boston Harbor Islands, the carabid portion of which was done in collaboration with Davidson and Bell. Other graduates of the Field Naturalist program who pursued entomological interests include Jeff Collins, Susan Morgan and Mark Ward.

Ross retired from UVM in 2000 but spent four subsequent summers teaching a field course in entomology at UVM. He continues his work with rhysodine beetles and many collaborators in Europe still send him specimens mainly from expeditions to China and SE Asia. Most recently, Ross has recognized specimens that David Kavanaugh collected in Yunnan as belonging to three new species of three rhysodine genera (Bell and Bell 2011). Ross continues scientific activity as Research Associate with the Carnegie Museum in Pittsburgh. Ross has now turned his attention to the perplexing matters of rhysodine zoogeography, hoping to find some sensible patterns in the wealth of new data that he and Joyce have generated. They have more than quadrupled our knowledge of rhysodines by adding description of c. 260 new species to the list of about 80 known when they started.

## Publications of RT Bell and JR Bell

Bell RT (1955) Species of the *pallipes* group of *Nebria* in eastern United States (Coleoptera, Carabidae). Proceedings of the Entomological Society of Washington 57: 265-267.

Bell RT (1957) *Carabus auratus* L. (Coleoptera, Carabidae) in North America. Proceedings of the Entomological Society of Washington59: 254.

Bell RT (1958) Intraspecific variation in *Chlaenius tomentosus* (Say) (Coleoptera, Carabidae). Annals of the Entomological Society of America 51: 432-435.

Bell RT (1959) A new species of *Scaphinotus* Dej., intermediate between *Scaphinotus* s. str. and *Irichroa* Newman (Coleoptera, Carabidae). Proceedings of the Entomological Society of Washington 61: 11-13.

Bell RT (1960) A revision of the genus *Chlaenius* Bonelli (Coleoptera, Carabidae) in North America. Miscellaneous Publications of the Entomological Society of America1: 97-166.

Bell, RT, Bell JR (1962) The taxonomic position of the Rhysodidae (Coleoptera). The Coleopterists Bulletin 16: 99-106.

Bell RT (1964) Does *Gehringia* belong to the Isochaeta? (Coleoptera: Carabidae). The Coleopterists Bulletin 18: 59-61.

Bell, RT, Bell JR (1964) *Platypatrobus lacustris* Darlington in Vermont (Coleoptera: Carabidae). Proceedings of the Entomological Society of Washington 66: 100.

Bell RT (1965) Coxal cavities and the phylogeny of the Adephaga. Proceedings of the XIIth International Congress of Entomology, pp. 80-81.

Bell RT (1966a) *Trachypachus* and the origin of the Hydradephaga (Coleoptera). The Coleopterists Bulletin 20: 107-112.

Bell RT (1966b) *Chlaenius patruelis* LeConte a valid species (Coleoptera: Carabidae). Proceedings of the Entomological Society of Washington 68: 321-322.

Bell RT (1967) Coxal cavities and the classification of the Adephaga (Coleoptera). Annals of the Entomological Society of America60: 101-107.

Bell RT (1970) The Rhysodini of North America, Central America, and the West Indies (Coleoptera: Carabidae or Rhysodidae). Miscellaneous Publications of the Entomological Society of America6: 289-324.

Bell RT (1971a) Carabidae (ground beetles). Grassland Biome, United States International Biological Program, Technical Report (Colorado State University, Fort Collins, Colorado) No. 66: 58 pages.

Bell RT (1971b) Handbook of the Malacostraca of Vermont and neighboring regions. Privately printed. 65 pages.

Bell RT (1973) A new species of *Clinidium* from Guatemala (Coleoptera, Carabidae or Rhysodidae). Proceedings of the Entomological Society of Washington 75: 279-282.

Bell RT (1975) *Omoglymmius* Ganglbauer, a separate genus (Coleoptera: Carabidae or Rhysodidae). The Coleopterists Bulletin 29: 351-352.

Bell RT Bell JR (1975) Two new taxa of *Clinidium* (Coleoptera: Rhysodidae or Carabidae) from eastern U. S., with a revised key to U. S. *Clinidium*. The Coleopterists Bulletin 29: 65-68.

Spence JR, Bell RT , Bell JR (1976) The larvae of *Nebria lacustris* (Casey) and *Nebria pallipes* (Say) (Coleoptera, Carabidae). The Coleopterists Bulletin 30: 81-84.

Bell RT (1977) Ergebnisse der Bhutan-Expedition 1972 des Naturhistorischen Museums in Basel Coleoptera: Fam. Rhysodidae. Entomologica Basiliensia 2: 151-158.

Davidson RL, Bell RT (1977) Note on the distribution and habitat of *Platynus opaculus* LeConte (Coleoptera: Carabidae) in Vermont. Cordulia 3: 157-158.

Bell RT (1978) The habitat of *Nebria suturalis* LeConte in Vermont (Coleoptera: Carabidae). Cordulia4: 82.

Bell RT, Bell JR (1978) Rhysodini of the world. Part I. A new classification of the tribe, and a synopsis of *Omoglymmius*, subgenus *Nitiglymmius*, new subgenus (Coleoptera: Carabidae or Rhysodidae). Quaestiones Entomologicae 14: 43-88.

Bell RT, Nielsen GR (1978) *Evarthrus sodalis* sodalis LeConte in Vermont (Coleoptera: Carabidae). Cordulia 4:

Bell RT, Bell JR (1979) Rhysodini of the world. Part II. Revisions of the smaller genera (Coleoptera: Carabidae or Rhysodidae). Quaestiones Entomologicae 15: 377-446.

Bell RT (1979) Zoogeography of Rhysodini--do beetles travel on driftwood? In: Erwin, TL, Ball GE, Whitehead DR, Halpern AL (Eds.). Carabid Beetles: their Evolution, Natural History and Classification. Proceedings of the First International Symposium of Carabidology, Smithsonian Institution, Washington, D. C., August 21, 23 and 25, 1976. Dr. W Junk bv Publishers, The Hague-Boston-London, 331-342.

Bell RT, Bell JR (1981) Insects of Micronesia, Coleoptera, Rhysodidae. Bernice P. Bishop Museum Insects of Micronesia 15(2): 51-67.

Bell RT, Bell JR (1982) Rhysodini of the world. Part III. Revision of *Omoglymmius* Ganglbauer (Coleoptera: Carabidae or Rhysodidae) and substitutions for preoccupied generic names. Quaestiones Entomologicae 18: 127-259.

Bell RT (1985a) A catalog of the Coleoptera of America north of Mexico. Family: Rhysodidae. U. S. Department of Agriculture, Agriculture Handbook 529-4, fascicle 4: x + 4 pages.

Bell RT (1985b) Zoogeography and ecology of New Guinea Rhysodini (Coleoptera: Carabidae). In: Ball, GE (Ed.). Taxonomy, Phylogeny and Zoogeography of Beetles and Ants. Dr. W. Junk Publishers, Dordrecht, 221-236.

Bell RT (1985c*) Pentagonica* of the West Indies (Coleoptera: Carabidae). The Coleopterists Bulletin 39: 321-327.

Bell RT, Bell JR (1985) Rhysodini of the world. Part IV. Revisions of *Rhyzodiastes* Fairmaire and *Clinidium* Kirby, with new species in other genera (Coleoptera: Carabidae or Rhysodidae). Quaestiones Entomologicae 21: 1-172.

Bell RT (1987) A new species of *Pentagonica* Schmidt-Goebel (Coleoptera: Carabidae) from the southwestern United States. The Coleopterists Bulletin 41: 373-376.

Bell RT, Bell JR (1987a) A new species of *Clinidium* Kirby (Coleoptera: Carabidae or Rhysodidae) from Mexico, and descriptions of the females of two Neotropical members of the genus. Annals of Carnegie Museum 56:193-196.

Bell RT, Bell JR (1987b) A new subtribe, genus and species of Rhysodini from South Africa (Coleoptera: Carabidae or Rhysodidae). Journal of the Entomological Society of Southern Africa 50: 287-290.

Bell RT, Bell JR (1987c) Rhysodine beetles in the Geneva collection: a new species of *Yamatosa*, and a major range extension for *Omoglymmius sakuraii* Nakane (Coleoptera: Carabidae or Rhysodidae). Revue suisse de Zoologie 94 : 683-686.

Bell RT, Davidson RL (1987) *Harpalus rubripes* (Duftschmid), a European ground beetle new to North America (Coleoptera: Carabidae). The Coleopterists Bulletin 41: 56.

Bell RT, Bell JR (1988) Rhysodini of Sulawesi and nearby islands (Coleoptera: Carabidae or Rhysodidae). Journal of the New York Entomological Society 96: 7-15.

Bell RT, Bell JR (1989a) Revision of the *chimbu* group of *Scopodes* (Coleoptera: Carabidae). The Coleopterists Bulletin 43: 157-161.

Bell RT, Bell JR (1989b) Rhysodine beetles in the Geneva collection II: new species of *Yamatosa* and *Omoglymmius*, descriptions of undescribed sexes in other species, and some major range extensions (Coleoptera: Carabidae or Rhysodidae). Revue suisse de Zoologie96 : 637-642.

Bell RT (1990) Chapter 35. Insecta: Coleoptera, Carabidae, Adults and Larvae. *In*: Dindal, DL (Ed.). Soil Biology Guide. John Wiley and Sons, New York. xx + 1,349 pages.

Bell RT (1991) Rhysodidae (Adephaga). Pages 304-305 *In*: Stehr, FW (Ed.). Immature Insects. Volume 2. Kendall/Hunt Publishing Co., Dubuque, Iowa, 1053-1092.

Bell RT, Bell JR (1991) The Rhysodini of Australia (Insecta: Coleoptera: Carabidae or Rhysodidae). Annals of Carnegie Museum 60: 179-210.

Bell RT, Hoebeke ER, Liebherr JK (1991) Revised distribution of the immigrant carabid *Bembidion obtusum* (Coleoptera: Carabidae) in eastern North America. Entomological News102: 174-178.

Bell RT (1992) The carabid fauna of the New England mountains (Coleoptera: Carabidae). *In*: Noonan, GR, GE Ball and NE Stork (Eds.). The Biogeography of Ground Beetles of Mountains and Islands. Intercept Ltd., Andover, United Kingdom, 43-52.

Bell RT, Bell JR (1993) Rhysodine beetles (Insecta: Coleoptera: Carabidae or Rhysodidae): new species, new data and revised keys to *Omoglymmius* (subgenera *Omoglymmius* and *Pyxiglymmius*). Annals of Carnegie Museum 62: 165-185.

Bell RT (1994) Beetles than cannot bite: functional morphology of the head of adult rhysodines (Coleoptera: Carabidae or Rhysodidae). The Canadian Entomologist126: 667-672. doi: 10.4039/Ent126667-3

Bell RT, Bell JR (1995) The Rhysodini (Insecta: Coleoptera: Carabidae) of Cuba. Annals of Carnegie Museum 64: 185-195.

Bell RT (1998) Where do the Rhysodini belong? *In*: Ball, GE, A Casale and A Vigna Taglianti (Eds.) Phylogeny and Classification of Caraboidea (Coleoptera: Adephaga). Proceedings of a Symposium (28 August, 1996, Florence, Italy) XX International Congress of Entomology. Atti, Museo regionale di Scienze naturale, Torino 261-272.

 Bell RT (1999) Rhysodini. Wrinkled Bark Beetles. Version 14 December 1999. http://tolweb.org/Rhysodini/67/1999.12.14. In: The Tree of Life Web Project, http://tolweb.org/

Leonard JG, Bell RT (1999) Northeastern Tiger Beetles: a field guide to the tiger beetles of New England and eastern Canada. CRC Press, Boca Raton, Florida. 176 pages.

Bell RT, Bell JR (2000) Rhysodine beetles (Insecta: Coleoptera: Carabidae or Rhysodidae): new species, new data II. Annals of Carnegie Museum 69:69-91.

Bell RT, Bell JR (2002) Two new species of Rhysodini (Coleoptera: Carabidae) with revised keys to *Yamatosa* Bell and Bell and *Omoglymmius* (*Pyxiglymmius*) Bell and Bell. Stuttgarter Beiträge zur Naturkunde, Serie A (Biologie), Nr. 636:1-7.

Bell RT (2003) Family Rhysodidae Laporte, 1840. In: Löbl, I, and A. Smetana (Eds.). Catalogue of Palaearctic Coleoptera. Volume I. Archostemata - Myxophaga - Adephaga. Apollo Books, Stenstrup, 78.

Bell RT, Bell JR (2009) Rhysodine beetles (Insecta: Coleoptera: Carabidae): new species, new data III. Annals of Carnegie Museum 78: 45-77. doi: 10.2992/007.078.0104

Bell, RT (2010) Les Rhysodini de Guyane (Coleoptera, Carabidae). Suppl. Bulletin de liason d’ACOREP-France “Les Coleopteriste”, Tome II: 46-49.

Bell RT, JR Bell (2011) Four new species of Rhysodini (Coleoptera: Carabidae) with revised keys to *Grouvellina* Bell & Bell and the *mishmicus* group of *Rhyzodiastes* Fairmaire. Stuttgarter Beiträge zur Naturkunde A, Neue Serie 4: 129-135.

